# Evaluating Chinese migrant workers’ housing conditions by diarrhea disease prevalence

**DOI:** 10.1057/s41271-025-00567-9

**Published:** 2025-05-03

**Authors:** Juntao Lyu, Baobin Feng, Hansoo Kim, Gayatri Marwah

**Affiliations:** 1 Cancer Health Services Research Unit, Centre for Health Policy, School of Population and Global Health, The University of Melbourne, Level 4, 207 Bouverie Street, Melbourne, VIC 3010 Australia; 2 Ministry of Emergency Management of China, National Institute of Natural Hazards, Beijing, China; 3 Griffith University, Gold Coast, Australia

**Keywords:** Chinese migrant workers, Diarrhea, Health inequalities, Housing, Multi-level logistic regression, Urban

## Abstract

Chinese migrant workers often face significant health-related social inequalities, particularly in housing, in urban China. However, there is limited research investigating the health impacts of housing inequalities among migrant workers. We examined the accommodation types associated with the prevalence of diarrhea among migrant workers in urban China. We used a nationwide survey data to investigate the overall housing conditions and applied multilevel logistic regression models to analyze the association between diarrhea and housing types. The findings highlight that the prevalence of diarrhea among migrant workers is significantly associated with housing types rather than neighborhood or income levels. Compared with living in private rental properties, migrants living in government-subsidized properties have significantly increased odds of reporting diarrhea episodes (OR = 1.41; 95% CI 1.23–1.61; p < 0.001). This study indicated the need to address the quality and maintenance of housing infrastructure rather than ownership status alone.

## Key messages

- Accommodation type is a key determinant of diarrhea prevalence among Chinese migrant workers, outweighing regional or income disparities.
- Migrant workers living in government-subsidized housing have a greater likelihood of diarrhea compared to those in private rentals, highlighting health challenges linked to the housing conditions.
- These findings suggest the need for public health policies to review and prioritize housing conditions, particularly in government-subsidized properties, to mitigate health risks for migrant populations in urban China.

## Introduction

Diarrhea is an infectious disease and a significant global health concern. In 2015, an estimated 2.39 billion episodes of diarrhea occurred globally. It was the eighth-leading cause of death among all ages and the fifth-leading cause of death among children younger than five years old, responsible for more than 1.6 million deaths in 2016 [1–3]. The Chinese incidence rate of dysentery, a type of gastroenteritis that results in bloody diarrhea, decreased from 38.3/100,000 in 2004 to 9.0/100,000 in 2016 nationwide [4]. Due to constrained healthcare resources, social support, and economic conditions, Chinese migrant workers often have a higher risk of infectious diseases such as diarrhea in urban China [5]. In 2017, more than half of Chinese migrant workers reported experiencing diarrhea [6], with significantly higher rates in Beijing and Tianjin compared to other regions in China [4].

In developing countries, housing factors, such as urban slums, are often considered the epicenters of diarrhea diseases [7]. For example, urban slums in India experience higher morbidity rates of diarrhea diseases due to overcrowded living conditions and inadequate water sanitation [7]. However, China and India have very different urban living spaces [8]. Migrant workers in urban China are mostly from rural or underdeveloped areas and are often institutionally and socially discriminated against in urban cities [9]. Unlike the defined urban slums of India, the living spaces of Chinese migrants are integrated into the general urban landscape [8]. This complexity is further compounded by multidimensional accommodation types within the same urban neighborhoods, ranging from local residences to semi-detached secondary housing [10, 11].

There have been attempts to investigate the inequalities presented through housing types among migrant workers in urban China. From a socioeconomic perspective, Wang and Li found that private properties were owned mainly by migrants with higher incomes and higher education who were more skilled and married [12]. However, health inequalities are more complex. Recent research conducted by Jia [13] found that Chinese migrant workers who are homeowners with mortgages are associated with a significantly higher risk of poorer health compared to those renting privately despite being ranked higher socioeconomically [13]. These findings demonstrate that socioeconomic and housing conditions are important for health outcomes related to diarrhea [14].

In urban China, the housing types for migrant workers range from private properties, rental properties (entire-rental and shared rental accommodations), and employment accommodations (onsite and off-site) [15], to government-subsidized security housing initiatives (purchased or rented properties) [16], and other informal accommodations. Detailed evidence to support more comprehensive and effective policies to tackle health inequalities in urban China is desirable. Examining different health outcomes across these various types of accommodations can provide a better understanding of the health inequalities that migrant workers face in urban China.

In this study we explored a cross-sectional association between diarrhea illnesses and accommodation types among Chinese migrant workers using the national survey data collected in 2017. We seek to identify the accommodation types with higher risks of diarrhea and address the health inequalities that Chinese migrant workers face in urban China.

## Data and methods

This cross-sectional study utilized the China Migrants Dynamic Survey (CMDS) 2017 database, administered by the Migrant Population Service Centre, National Health Commission P. R. China. This database covers 31 mainland China provinces and 12 months between March 2016 and March 2017 [17]. As a rural-to-urban migrants-focused survey, the participants are limited to migrants over 15 years old living in the cities outside their family registration region for over one month. There are 169,989 participants in total, and the participants were sampled by the multi-stage probability proportionate to size sampling method according to the population sizes at five administrative levels—provinces, cities, districts, townships, and villages.

**Fig. 1:**
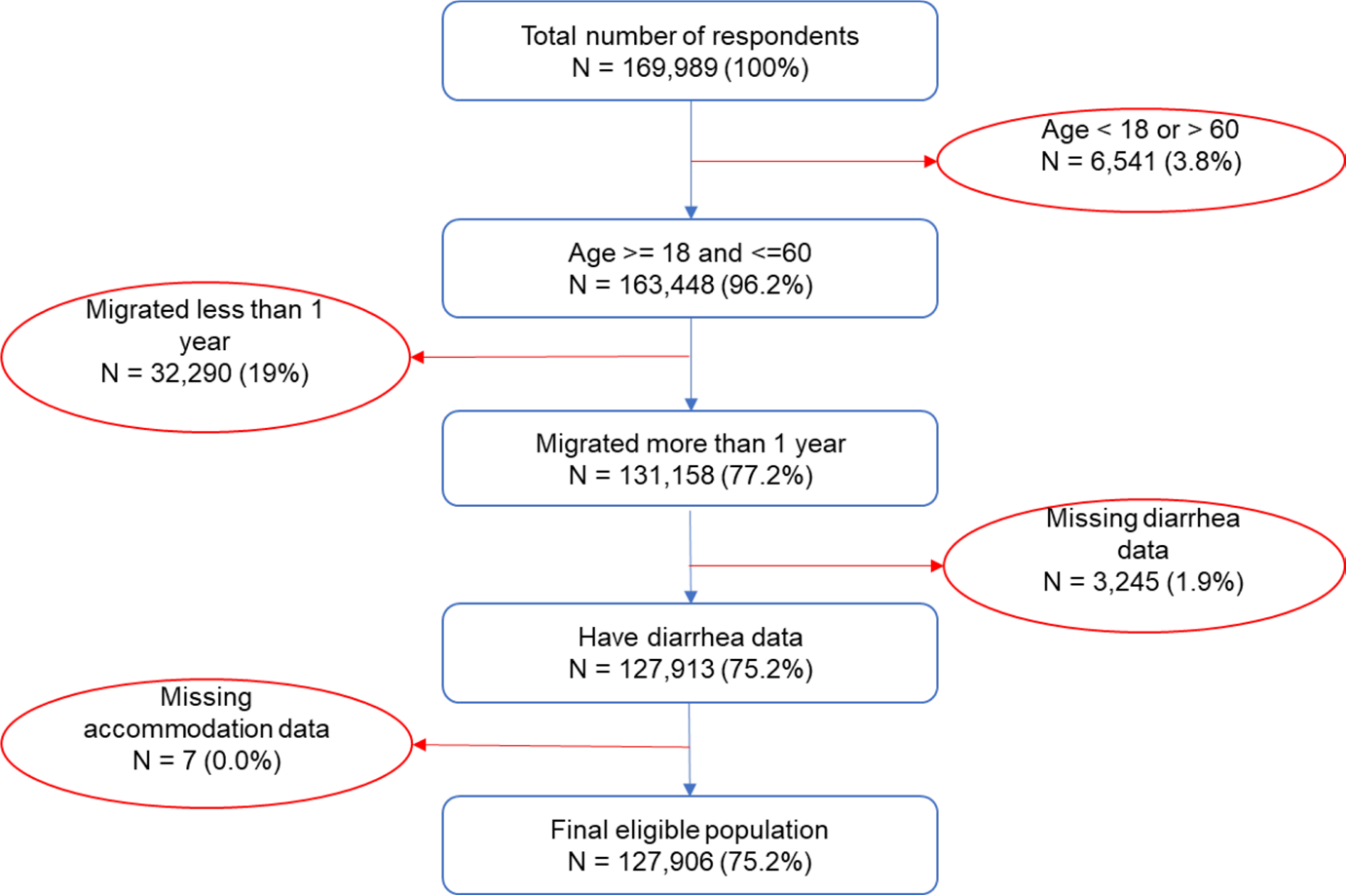
Flow chart of the sample selection process

Eligible samples in this study were selected based on the following requirements: (1) Migrant workers who have lived in current urban cities for more than one year, aged between 18 and 60; and (2) Migrant workers have demographic, diarrhea, and accommodation data available. Figure 1 shows the flow chart of the selection process against these criteria.

Approximately 19% of the respondents were excluded because their migration duration was less than one year during the survey. This criterion was chosen to ensure that this study’s population was settled in urban accommodations for more than one year, not just seasonal workers. It also ensures that the reported diarrhea episodes in that survey were associated with migrant workers’ urban accommodations. The age criterion was chosen to exclude potentially vulnerable age groups (3.8% excluded), and the missing data criterion was chosen to ensure the data quality (1.9% excluded). The final sample size was 127,906 out of 169,989 observations, located in 351 urban cities, which include 1290 lower-level administrative districts.

### Outcome and exposure variables

The outcome variable is self-reported diarrhea (the binary answers of yes and no were collected and coded as 1 and 0). Self-reported diarrhea is widely used in developing countries to reflect foodborne diseases in social epidemiological research [18]. In the CMDS questionnaire, participants were asked if they had experienced diarrhea disease at least once during the last 12 months. The survey defines diarrhea disease as having at least three episodes of diarrhea in a day. This definition is relatively clear since it is quantified, easy to understand, and less likely to be affected by subjective understandings.

Demographic variables such as age, sex, marital status, family income per person, education levels, and migration (cross-province or inner province) as independent factors at the individual level were considered.

The accommodation types were classified by the CMDS team in their national survey questionnaire. The classification considered migrant workers’ homeownership through the private real estate market or government subsidies, accommodation-related employment benefits, and other informal living arrangements [19] (see Table 1).

**Table 1:** Chinese migrant workers’ accommodation types

Category	Type	Description
Self-owned	Private property (owning)	Properties purchased from formal real estate markets and shared within the same households
	Government-subsidized property	Properties purchased from government-subsidized projects and shared within the same households
	Village property	Properties purchased from remote urban areas without full ownership
Rental	Private entire-rental	Properties rented for self or own family
	Private shared rental	Properties rented with non-family others
	Government-subsidized rental	Properties rented with government subsidies
Employment provided	Employment accommodation—offsite	Accommodations provided by employers, separated from worksites, such as the dormitory rooms beside the factories
	Employment accommodation—onsite	Accommodations provided by employers, within worksites, such as the construction workers who live in temporary blocks within construction sites
Other informal types	Self-built	Informal accommodations in remote outskirt areas built by migrant workers [20]
	Other temporary accommodation	Other types of informal and temporary accommodations

### Statistical analysis

We examined whether diarrhea prevalence is associated with the accommodation types among Chinese migrant workers across different urban cities in China. We clustered the residential areas into three levels. The first level categorized all individuals into China's eastern, western, and central regions. These three regions divide all cities in Mainland China based on geographic location and socioeconomic status [21]. The east region is the most developed, consisting of 11 coastal provinces. The central region, including nine inland provinces, is less developed. The western region is China’s most underdeveloped area, covering ten provinces. The second level clusters 1290 urban districts. The 1290 urban districts were categorized into two types: higher-income and lower-income districts. We defined the higher-income district based on the percentage of residing migrants with higher incomes. Migrants with an average family income per person exceeding 1870 Chinese Yuan per month were classified as having higher incomes, whereas those below this threshold were categorized as having lower incomes [22]. A district is designated as a higher-income district if more than 50% of its migrant population falls into the higher-income category. The third level is the individual level, which contains the types of accommodations and other individual characteristics.

We applied the multilevel logistic regression method following the three-step procedure to inspect the impact of different areas and broader sociodemographic factors [23]. We analyzed the relationship between accommodation types and diarrhea outcomes using three logistic regression models, with accommodation types as the independent variables and diarrhea outcomes as the dependent variable. The dataset included 127,906 observations clustered across three regions and 1290 urban districts. Private entire-rental accommodation, the most prevalent housing type among migrants in urban China, was used as the reference category. All other accommodation types were compared to private entire-rental housing to assess their impact on reported diarrhea outcomes.

The base Model 1 estimated the variation of diarrhea outcomes between different regions and urban districts without individual factors. We tested the regional differences, as well as the different districts nested in these three regions. Model 1 established the base of area differences for this analysis:

$$Logit\left({P}_{i}\right)=log\left(\frac{{P}_{i}}{1-{p}_{i}}\right)=M+{E}_{A },$$

where M represents the overall mean probability on the logistic scale, and ${{\varvec{E}}}_{{\varvec{A}}}$ represents the area-level residuals on the logistic scale.

Continuing from the regional analysis in Model 1, Model 2 introduced individual-level characteristics, including age, sex, income, education, and accommodation types. Model 2 aims to identify the most influential socio-demographic factors affecting diarrhea prevalence among migrants:

$$Logit\left({P}_{i}\right)=M+{\beta }_{1}Se{x}_{i}+{\beta }_{2}Ag{e}_{i}+{\beta }_{3}Marriag{e}_{i}+{\beta }_{4}Migratio{n}_{i}+{\beta }_{5}Incom{e}_{i}+{\beta }_{6}Educatio{n}_{i}+{\beta }_{7}Accommodatio{n}_{i}+{E}_{A} ,$$

where *β*_*1*_*, β*_*2*_*…, β*_*7*_ are the individual covariate regression coefficients.

Model 3 is the multilevel logistic regression model with individual-level and area variables to reveal the impact of area differences and individual characteristics across areas by including the area variables:

$$Logit\left({P}_{i}\right)=M+{\beta }_{1}Se{x}_{i}+{\beta }_{2}Ag{e}_{i}+{\beta }_{3}Marriag{e}_{i}+{\beta }_{4}Migratio{n}_{i}+{\beta }_{5}Incom{e}_{i}+{\beta }_{6}Educatio{n}_{i}+{\beta }_{7}Accommodatio{n}_{i}+{\beta }_{8}Incom{e}_{distric{t}_{i}}+{E}_{A} ,$$

where the additional *β*_*8*_ is the regression coefficient for the district income level variable. For Model 3, we generated the intraclass correlation coefficient (ICC), demonstrating the proportion of the total variance in the outcome attributed to the district level.

$${\text{ICC }} = {\text{ VA}}/\left( {{\text{VA}} + {\text{VI}}} \right),$$

where VA is the district-level variance, and VI is the individual-level variance. We also generated the marginal R^2^ and the conditional R^2^ to assess the goodness of fit of Model 3. Model 2 and Model 3 are constructed to evaluate how individual and area-level variables interplay to affect health outcomes and depict how each variable contributes to the model, with coefficients representing the strength and direction of these relationships.

The multilevel logistic regression models were performed using R (4.3.3) and RStudio (2023.12.1 + 402) [24, 25].

## Results

According to the CMDS data, in 2017, most migrants in urban China were between 18 and 40 years of age (91%), married (86%), and lived in private rental accommodations (55%), including entire-rental (46%) and shared rental (9%) accommodations. Most homeowners owned private properties by purchasing from private markets (24%), and only a small portion of the migrants were granted government subsidies for their accommodations (2%). In terms of diarrhea prevalence, 14.5% of migrant workers reported having experienced at least one time of diarrhea disease, and 85.5% reported no experience of diarrhea disease. Table 2 summarizes the characteristics of migrant workers who did and did not report experiencing diarrhea over the 12 months in 2017. Each demographic group was divided into two groups: reported diarrhea and did not report diarrhea. A higher proportion reported having diarrhea were in Western China, among males, young (18–30 years), married respondents, people who migrated within provinces, who had relatively higher income, were low educated, and lived in private entire-rental properties.

**Table 2:** Comparison of migrants’ characteristics between those who reported diarrhea and those who did not report diarrhea

	Did not report diarrhea 85.5% (n = 109,362)	Reported diarrhea 14.5% (n = 18,544)	p-value
Regions			
Western	37.1% (40,596)	41.2% (7647)	< 0.001
Middle	22.2% (24,244)	21.1% (3922)	
Eastern	40.7% (44,522)	37.6% (6975)	
Sex			
Male	50.9% (55,617)	54.7% (10,146)	< 0.001
Female	49.1% (53,745)	45.3% (8398)	
Age group			
18 ~ 30	30.4% (33,253)	36.5% (6764)	< 0.001
31 ~ 40	34.7% (37,962)	34.1% (6331)	
41 ~ 50	26.0% (28,483)	22.0% (4080)	
51 ~ 60	8.8% (9664)	7.4% (1369)	
Marital status			
Single	13.9% (15,245)	14.8% (2741)	< 0.001
Married	86.1% (94,117)	85.2% (15,803)	
Migration			
Inter-provincial	50.9% (55,696)	53.5% (9921)	< 0.001
Inner-provincial	49.1% (53,666)	46.5% (8623)	
Income			
Lower	44.9% (49,096)	44.7% (8298)	0.720
Higher	55.1% (60,266)	55.3% (10,246)	
Education			
High school or college degrees	38.8% (42,407)	42.3% (7835)	< 0.001
Below high school	61.2% (66,955)	57.7% (10,709)	
Accommodation types			
Private rental (entire-rental)	46.4% (50,743)	43.0% (7972)	< 0.001
Private property (owning)	23.4% (25,558)	25.3% (4696)	
Private rental (shared rental)	9.5% (10,426)	9.1% (1681)	
Employment accommodation—offsite	7.5% (8224)	7.4% (1364)	
Self-built	3.8% (4128)	4.8% (895)	
Village property (owning)	2.9% (3136)	3.1% (566)	
Employment accommodation—onsite	2.3% (2522)	2.5% (457)	
Other temporary accommodation	1.9% (2066)	2.0% (364)	
Government-subsidized property (owning)	1.3% (1442)	1.8% (325)	
Government-subsidized rental	1.0% (1117)	1.2% (224)	
Area variable: residing district			
Higher-income districts	63.3% (69, 259)	60.3% (11, 190)	< 0.001
Lower-income districts	36.7% (40, 103)	39.7% (7, 354)	

**Fig. 2:**
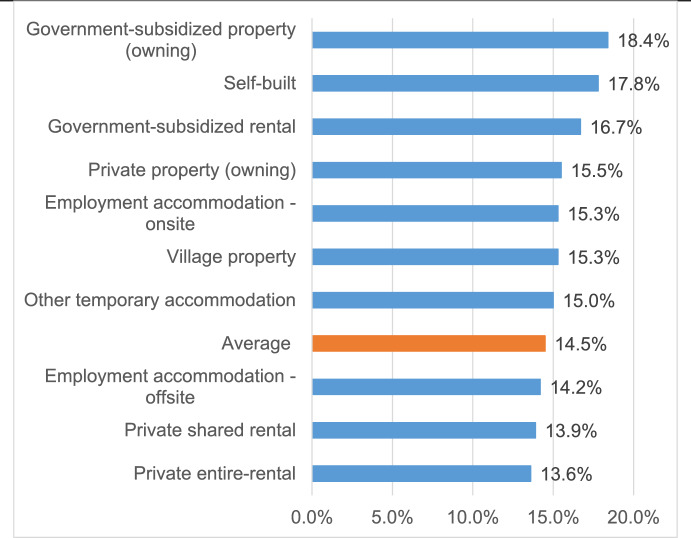
Proportional prevalence of diarrhea episodes among migrant workers by accommodation type compared to the average level

Figure 2 illustrates the prevalence of diarrhea episodes among migrant workers across various accommodation types, presented as proportions relative to the average prevalence of 14.5%. The highest reported prevalence of diarrhea was observed among residents in government-subsidized properties, followed by those in self-built properties, government-subsidized rental properties, private properties, village properties, onsite employment accommodations, and other informal temporary housing. These accommodation types are associated with a higher proportion of residing migrants who reported above-average levels of diarrhea episodes. In contrast, the lowest prevalence was reported in the private entire-rental group (13.6%), followed by the private shared-rental group (13.9%) and offsite employment accommodations (14.2%), all of which had below-average levels of reported diarrhea episodes.

Table 3 presents the outcomes of the three multi-level logistic regression models. The Akaike Information Criterion (AIC) was used to assess model fit, with lower AIC values indicating a better trade-off between goodness of fit and model complexity. Model 1 had an AIC of 105,883, Model 2 had an AIC of 103,502, and Model 3 had an AIC of 103,535. Although Model 3 added an area variable, its AIC (103,535) was slightly higher than Model 2 (103,502), suggesting that the additional area variable did not substantially improve the model’s fit.

**Table 3:** Comparison of the three logistic regression models

	Model 1	Model 2		Model 3	
		OR (95% CI)	p-value	OR (95% CI)	p-value
Individual level variables					
Female (vs. male)		0.81 (0.79–0.84)	** < 0.001**	0.82 (0.79–0.84)	** < 0.001**
Married (vs. single)		1.05 (1.00–1.11)	**0.038**	1.05 (1.00–1.11)	**0.038**
Inter-province migration (vs. inner province)		0.98 (0.94–1.02)	0.235	0.98 (0.94–1.02)	0.237
Lower income (vs. higher income)		0.99 (0.96–1.03)	0.640	0.99 (0.96–1.03)	0.632
Higher education (vs. below high school)		1.08 (1.04–1.12)	** < 0.001**	1.08 (1.04–1.12)	** < 0.001**
Age group 31–40 (vs 18–30)		0.79 (0.76–0.83)	** < 0.001**	0.79 (0.76–0.83)	** < 0.001**
Age group 41–50 (vs 18–30)		0.68 (0.65–0.71)	** < 0.001**	0.68 (0.65–0.71)	** < 0.001**
Age group 51–60 (vs 18–30)		0.65 (0.61–0.70)	** < 0.001**	0.65 (0.61–0.70)	** < 0.001**
Accommodation types (vs. private entire-rental)					
Private property (owning)		1.12 (1.07–1.17)	** < 0.001**	1.12 (1.07–1.17)	** < 0.001**
Private rental (shared rental)		1.03 (0.97–1.10)	0.288	1.03 (0.97–1.10)	0.288
Employment accommodation—offsite		1.01 (0.94–1.08)	0.839	1.01 (0.94–1.08)	0.837
Self-built		1.21 (1.11–1.32)	** < 0.001**	1.21 (1.11–1.33)	** < 0.001**
Village property (owning)		1.13 (1.02–1.24)	**0.019**	1.13 (1.02–1.24)	**0.017**
Employment accommodation—onsite		1.08 (0.97–1.21)	0.163	1.08 (0.97–1.21)	0.163
Other temporary accommodation		1.11 (0.98–1.25)	0.091	1.11 (0.98–1.25)	0.091
Government-subsidized property (owning)		1.41 (1.23–1.61)	** < 0.001**	1.41 (1.23–1.61)	** < 0.001**
Government-subsidized rental		1.16 (0.98–1.37)	0.075	1.17 (0.99–1.38)	0.075
Area level variable					
Lower-income District (vs higher-income district)				1.01 (0.93–1.09)	0.901
Measures of variation or clustering					
Region	N = 3	N = 3		N = 3	
District	N = 1290	N = 1290		N = 1290	
Regional variance	0.01	0.00		0.00	
District level variance	0.31	0.30		0.30	
ICC	0.09	0.09		0.09	
Marginal R^2^	0	0.011		0.011	
Conditional R^2^	0.087	0.096		0.096	
AIC	105,833	**103,502**		103,535	

Model 1 suggested that only 9% (ICC = 0.09) of the variance in diarrhea prevalence is associated with regions and districts, which is relatively low compared to the total variation in the data. In Model 2, individual characteristic variables significantly improved the model's explanatory power. The conditional R^2^ increased from 0.087 in Model 1 to 0.096 in Model 2. This indicates a better model fit and a more accurate data representation. Model 2 outcomes also suggested that the differences in individual gender, education level, age, and accommodation types are significantly associated with the prevalence of diarrhea among migrant workers in urban China. However, the family income and migration distance (cross-province or inner province) were insignificant in predicting diarrhea prevalence. The added area variable in Model 3 did not change the outcomes from Model 2, suggesting that the district stratified by their average income was also insignificant for the diarrhea prevalence outcomes. The regional variable and district variance remain relatively small. This suggested that accommodation types, rather than district locations, accounted for more of the variability of the prevalence of diarrhea in urban China.

The logistic regression outcomes highlighted that people living in government-subsidized properties and self-built accommodations had a higher likelihood of diarrhea diseases. Migrants residing in government-subsidized properties demonstrated the highest likelihood of reporting diarrhea episodes, with an odds ratio (OR) of 1.41 [95% confidence interval (CI) 1.23–1.61; p < 0.001], which is significantly higher compared to those living in the entire-rental type of accommodations. This was followed by migrant workers living in self-built properties (OR = 1.21; 95% CI 1.11–1.32; p < 0.001), those owning village properties (OR = 1.13; 95% CI 1.02–1.24; p = 0.019) and those owning private properties (OR = 1.12; 95% CI 1.07–1.17; p < 0.001), which also exhibited higher odds of reporting diarrhea episodes compared to those living in entire-rental accommodations.

## Discussion

This study demonstrated an association between housing inequalities and health disparities among Chinese migrant workers in the China Migrants Dynamic Survey. The findings highlighted that government-subsidized property residents have 1.41 times increased odds of diarrhea episodes than private rental accommodations. From a statistical perspective, we recognize that the large sample size of this study increases the likelihood that even small differences may achieve statistical significance. To address this, we reported all relevant coefficients to provide a comprehensive understanding of the findings while allowing interpretation of these differences beyond reliance on P-values alone. We are also aware that interactions between individual income and housing type may exist, particularly given the potential for these variables to influence diarrhea prevalence jointly. However, such interactions are likely diverse and depend on additional demographic factors, such as family size and age group, which were not the primary focus of this study. Furthermore, including area-level income as a contextual variable helps capture broader socioeconomic influences, reducing the necessity for explicitly testing interactions between individual income and housing type. By focusing on the main effects of housing type, this study aimed to provide a clear and interpretable understanding of the factors associated with diarrhea prevalence. Future studies could further investigate these interactions in specific subgroups with additional demographic and behavioral covariates.

We also noticed that the classification of accommodation types for migrant workers is more diverse than the categories listed in the CMDS questionnaire. For example, mortgages could be one reason private homeowners were associated with poorer health outcomes [13]. Location is another important factor since most government-subsidized properties are in remote city fringe areas, which increases the commute distances for dwellers [14]. The accuracy of our study's findings could be improved with access to more detailed information about accommodation characteristics. For example, the direct causes of diarrhea episodes, such as food, water, or other environmental factors. More epidemiologic data is required for future studies to reveal the various direct causes of the prevalence of diarrhea across different types of accommodations among migrant workers in urban China.

This study also has several inherent limitations. First, the cross-sectional design limits the ability to establish causality between exposure and outcome variables. Second, migrant workers may have relocated residences during the 12-month period, and the survey did not capture this information. Third, self-reported diarrhea may be inaccurate due to potential underreporting influenced by respondents’ health literacy. Additionally, detailed occupational information was not collected, preventing analysis of potential health impacts related to working conditions.

The prevalence of diarrhea among migrant workers can also be influenced by a range of other factors, which this study did not directly assess. Causes, such as stress, dietary changes, and chronic gastrointestinal conditions, may contribute to the observed disparities across housing types. For instance, stress associated with financial strain, overcrowding, or inadequate housing facilities may exacerbate gastrointestinal symptoms [26]. Similarly, the dietary habits of migrants, which might include increased reliance on low-cost, processed, or unsafe food, could interact with housing conditions to influence health outcomes [27, 28]. Future research should aim to disentangle the relative contributions of infectious and non-infectious causes to better understand the complex pathways linking housing conditions to diarrhea prevalence.

## Conclusions

In conclusion, this study highlighted the critical role of accommodation types as a key determinant of diarrhea prevalence among migrant workers in urban China, surpassing regional and income-related factors. Our findings revealed that government-subsidized housing and private homeownership, including self-built and privately purchased properties, are associated with an elevated risk of diarrhea prevalence. These results challenged the assumption that homeownership inherently confers health benefits [29]. The new findings highlight the urgent need to address the quality and maintenance of housing infrastructure rather than focusing solely on ownership status. Public health policies in urban China should prioritize improving the infrastructure of subsidized housing and addressing deficiencies in the private housing sector to mitigate health risks for vulnerable migrant populations. Addressing these gaps will enable public health initiatives to better support migrant communities and promote equitable health outcomes in urban China.

## Data Availability

This study utilized the administrative database ‘China Migrants Dynamic Survey 2017,’ provided by the Migrant Population Service Centre, National Health Commission, P.R. China. Dr. Baobin Feng applied for access to the data in April 2021, and the application was approved in the same month. Access to the database is restricted and can only be requested directly from the database administrator. All relevant analysis outcomes derived from the data are presented in the paper.
